# Making room for opinions

**DOI:** 10.1371/journal.pgen.1008015

**Published:** 2019-02-28

**Authors:** Gregory P. Copenhaver, Gregory S. Barsh

**Affiliations:** 1 Department of Biology and the Integrative Program for Biological and Genome Sciences, University of North Carolina at Chapel Hill, Chapel Hill, North Carolina, United States of America; 2 HudsonAlpha Institute for Biotechnology, Huntsville, Alabama, Department of Genetics, Stanford University School of Medicine, Stanford, California, United States of America

The British philosopher and social critic, Bertrand Russell, said “Do not fear to be eccentric in opinion, for every opinion now accepted was once eccentric” [1]. While the object of science is to reveal truth and describe the natural world by seeking objective facts, the path towards truth is not always apparent, and opinions matter. Which experimental approach and/or system will be most effective? How is a phenotype of biological or medical interest best captured analytically? And, to what extent can the conclusions based on a model system be generalized to natural populations? It is at these intersections on the path towards truth that opinions are crucial. Opinions can inspire healthy, productive debate, which in turn can prompt us to scrutinize our knowledge base and ultimately seek a deeper understanding, or perchance to change our minds. And, while opinions should always be based on facts, opinions are dramatically distinct from facts. They are a reflection of the individual and are inherently biased. As editors and scientists, we believe that both factual reporting and expression of opinion are vital components in our efforts to advance science. In that spirit, *PLOS Genetics* is initiating a new Opinion Pieces series with an inaugural article entitled “Outside In”, in which Jonathan Flint explores an intersection on the path towards understanding behavior, and expresses his opinion about genetics and neurophysiology [2].

*PLOS Genetics* Opinion Pieces are not intended to be a platform for highlighting or critiquing individual studies (our Viewpoints, Perspectives and Formal Comments are better suited for those purposes). Instead, Opinion articles should be synoptic in scope, and focus on major trends, or lack thereof, in the field of genetics or on issues or problems that are of concern to the broader genetics community. We anticipate that these opinions will be frank, and at times even uncomfortable, but we also expect they will play a constructive role in enhancing transparency about how science is done, its strengths and shortcomings, and how it is interpreted. Importantly, the opinions expressed in these articles are those of their authors, and not necessarily shared by our editors or by PLOS. They will be editorially evaluated, but just as in traditional news journalism, we will use a different, more permissive, set of standards for Opinion Pieces as compared to Research articles.

We hope that you share our excitement about this new mode of communicating science to our readers and we enthusiastically welcome any feedback you may have.
